# Case report: whole exome sequencing of primary cardiac angiosarcoma highlights potential for targeted therapies

**DOI:** 10.1186/s12885-016-3000-z

**Published:** 2017-01-05

**Authors:** Leah Zhrebker, Irene Cherni, Lara M. Gross, Margaret M. Hinshelwood, Merrick Reese, Jessica Aldrich, Joseph M. Guileyardo, William C. Roberts, David Craig, Daniel D. Von Hoff, Robert G. Mennel, John D. Carpten

**Affiliations:** 1 Baylor Charles A. Sammons Cancer Center at Dallas, Baylor University Medical Center at Dallas, 3410 Worth Street, Dallas, TX 75246 USA; 2 Department of Internal Medicine, Baylor University Medical Center at Dallas, 3500 Gaston Ave, Dallas, TX 75246 USA; 3 Integrative Cancer Genomics, Translational Genomics Research Institute, 445N 5th Street, Phoenix, AZ 85004 USA; 4 Texas Oncology/US Oncology, 3410 Worth Street, Dallas, TX 75246 USA; 5 Anatomic Pathology and Clinical Pathology, Baylor University Medical Center at Dallas, 3600 Gaston Ave, Dallas, TX 75246 USA; 6 Clinical Translational Research Division Translational Genomics Research Institute, 445N 5th Street, Phoenix, AZ 85004 USA; 7 College of Medicine, Texas A&M Health Sciences Center, 3410 Worth Street, Dallas, TX 75246 USA

**Keywords:** Cardiac angiosarcoma, Whole exome sequencing, Activating gene mutation, Targeted therapies

## Abstract

**Background:**

Primary cardiac angiosarcomas are rare, but they are the most aggressive type of primary cardiac neoplasms. When patients do present, it is with advanced pulmonary and/or cardiac symptoms. Therefore, many times the correct diagnosis is not made at the time of initial presentation. These patients have metastatic disease and the vast majority of these patients die within a few months after diagnosis. Currently the treatment choices are limited and there are no targeted therapies available.

**Case presentation:**

A 56-year-old male presented with shortness of breath, night sweats, and productive cough for a month. Workup revealed pericardial effusion and multiple bilateral pulmonary nodules suspicious for metastatic disease. Transthoracic echocardiogram showed a large pericardial effusion and a large mass in the base of the right atrium. Results of biopsy of bilateral lung nodules established a diagnosis of primary cardiac angiosarcoma. Aggressive pulmonary disease caused rapid deterioration; the patient went on hospice and subsequently died. Whole exome sequencing of the patient’s postmortem tumor revealed a novel *KDR* (G681R) mutation, and focal high-level amplification at chromosome 1q encompassing *MDM4*, a negative regulator of TP53.

**Conclusion:**

Mutations in *KDR* have been reported previously in angiosarcomas. Previous studies also demonstrated that *KDR* mutants with constitutive KDR activation could be inhibited with specific KDR inhibitors in vitro. Thus, patients harboring activating *KDR* mutations could be candidates for treatment with KDR-specific inhibitors.

**Electronic supplementary material:**

The online version of this article (doi:10.1186/s12885-016-3000-z) contains supplementary material, which is available to authorized users.

## Background

Primary cardiac tumors are very rare; the reported incidence in most autopsy and surgery series varies from 0.001 to 0.3% [1–3]. Of tumors that arise in the heart, most are benign and approximately 25% are malignant. About 95% of the malignant tumors are sarcomas, and they comprise 10 to 15% of the total primary cardiac tumors. Sarcomas can develop in any site in the heart, but angiosarcomas, which account for a third of all sarcomas, normally originate in the right atrium near the atrioventricular groove [4, 5].

Initially, there are no noticeable symptoms for patients with cardiac angiosarcoma. When patients do present they tend to have locally advanced or metastatic disease. Most symptoms are related to intracardiac flow obstruction, pericardial effusion and tamponade, tumor embolism, and systemic or constitutional symptoms due to metastatic disease [6–8]. Lung metastases are common at presentation [4, 9]. Additionally, metastases have been reported in liver, lymph nodes, bone, adrenal glands, and to a lesser extent the central nervous system (CNS) and spleen [4, 9]. Due to the late stage at presentation, prognosis for patients with primary angiosarcoma of the heart is poor; median overall survival varies from 6 months or less for untreated patients to 12 months for patients with incomplete tumor resection [6, 10–12]. Tumor often invades into the adjacent tissues, making complete resection challenging, if not impossible. In some cases, cardiac transplantation for patients with primary cardiac angiosarcoma has been reported, albeit with poor outcomes, with most patients dying from recurrence within 1 year of surgery. Cardiac transplantation is not currently recommended even for patients without evidence of metastatic disease due to lack of survival benefit to patients with transplantation [13–15].

Existing treatment strategies include resection for non-metastatic disease, chemotherapy, and radiation. There are limited data showing chemotherapy treatment algorithms for cardiac angiosarcoma. Currently used chemotherapies include traditional agents such as paclitaxel, docetaxel, and doxorubicin. Angiogenesis inhibitors, drugs that target the growth of endothelial cells, such as bevacizumab, sunitinib, and sorafenib, have been used as well. Although some therapies may extend the lifespan of patients when combined with other modalities [16], none of the traditional chemotherapies or newer agents have been shown to eradicate microscopic disease [17–20]. Lack of efficacy in existing treatment options highlights the need for targeted cancer therapies for patients with cardiac angiosarcoma.

To date, various studies investigated specific genes and pathways altered in angiosarcoma. V-Myc Avian Myelocytomatosis Viral Oncogene Homolog (*MYC*) amplified tumors are common in radiation-induced and lymphedema-associated angiosarcomas [21]. Moreover, Fms Related Tyrosine Kinase 4 (*FLT4*) and *MYC* co-amplification was observed in 25% of secondary angiosarcomas [22]. Point mutations in V-Ki-ras2 Kirsten rat sarcoma viral oncogene homolog (*KRAS*) oncogene were found in liver angiosarcoma [23]. In other reports, a primary skin angiosarcoma and liver angiosarcoma lacked expression of p16, the product of the Cyclin Dependent Kinase Inhibitor 2A (*CDKN2A*) gene [24–26]. A number of studies found high expression of mutated Tumor protein P53 (*TP53*) gene in the tumor cells of angiosarcoma from various tissues of origin [26–29]. Microarray expression studies revealed upregulation of a number of vascular-specific receptor tyrosine kinases, including Tyrosine Kinase with Immunoglobulin like and EGF Like Domains 1 (*TIE1*), Kinase Insert Domain Receptor (*KDR*), SNF Related Kinase (*SNRK*), TEK Receptor Tyrosine Kinase (*TEK*), and Fms Related Tyrosine Kinase 1 (*FLT1*) in angiosarcomas [30]. Further examination of these five genes by full-sequencing identified 10% of angiosarcoma patients harbored mutations in the *KDR* gene [30]. More recently, whole exome sequencing of primary and secondary angiosarcoma demonstrated mutations in the endothelial phosphatase, Protein tyrosine phosphatase, receptor type, B (*PTPRB*) and Phospholipase C, gamma 1 (*PLCG1*), a signal transducer of tyrosine kinase activators [31]. In the current study, we used custom-designed expanded exome capture to interrogate the genome of an aggressive primary cardiac angiosarcoma case using a genome-wide sequencing approach that included analysis of the annotated coding exome along with additional probes that allowed for genome-wide copy number analysis and the structural analysis of specific regions of the genome to capture translocations and inversions. This analysis allowed for a comprehensive assessment of the genomic landscape of this aggressive angiosarcoma.

Herein we describe the results of whole exome sequencing a primary, cardiac angiosarcoma. We found a novel *KDR* (G681R) mutation which is a putative ligand-independent activating mutation. Additionally, we discovered a focal high-level amplification at chromosome 1q encompassing MDM4 p53 binding protein homolog (*MDM4*), a negative regulator of TP53. We discuss potential therapeutic therapies which may be effective in treating patients with tumors containing these mutations/amplifications.

## Case presentation

**Fig. 1:**
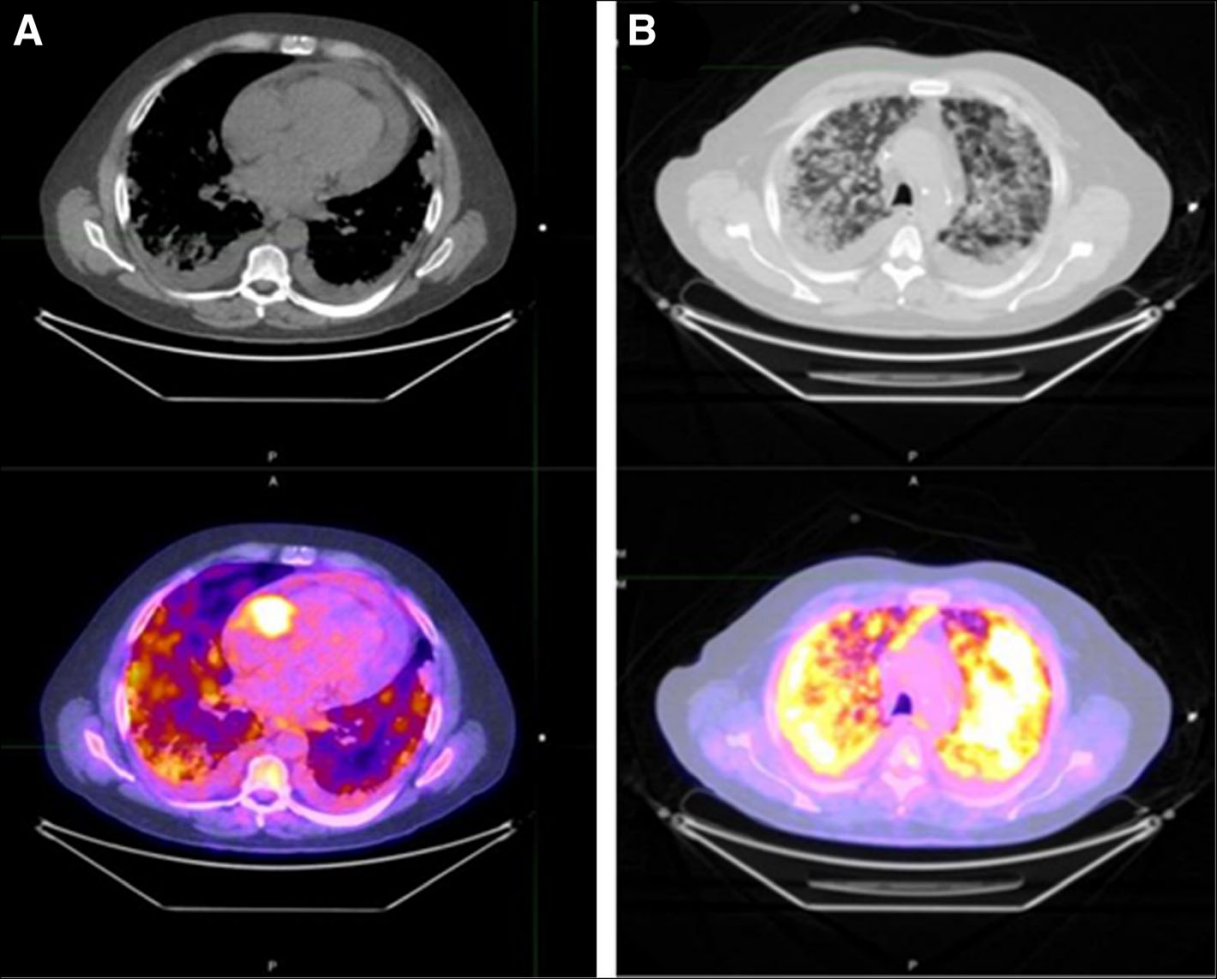
Chest PET/CT Scan*.* **a** Chest PET/CT scan of the chest revealed abnormal uptake in the right atrium with an SUV of 14.9. **b** Chest PET/CT scan showed ground glass appearance and uptake indicative of nodules and pleural effusions in lungs; maximal SUVs of lung nodules were 9.0

**Fig. 2:**
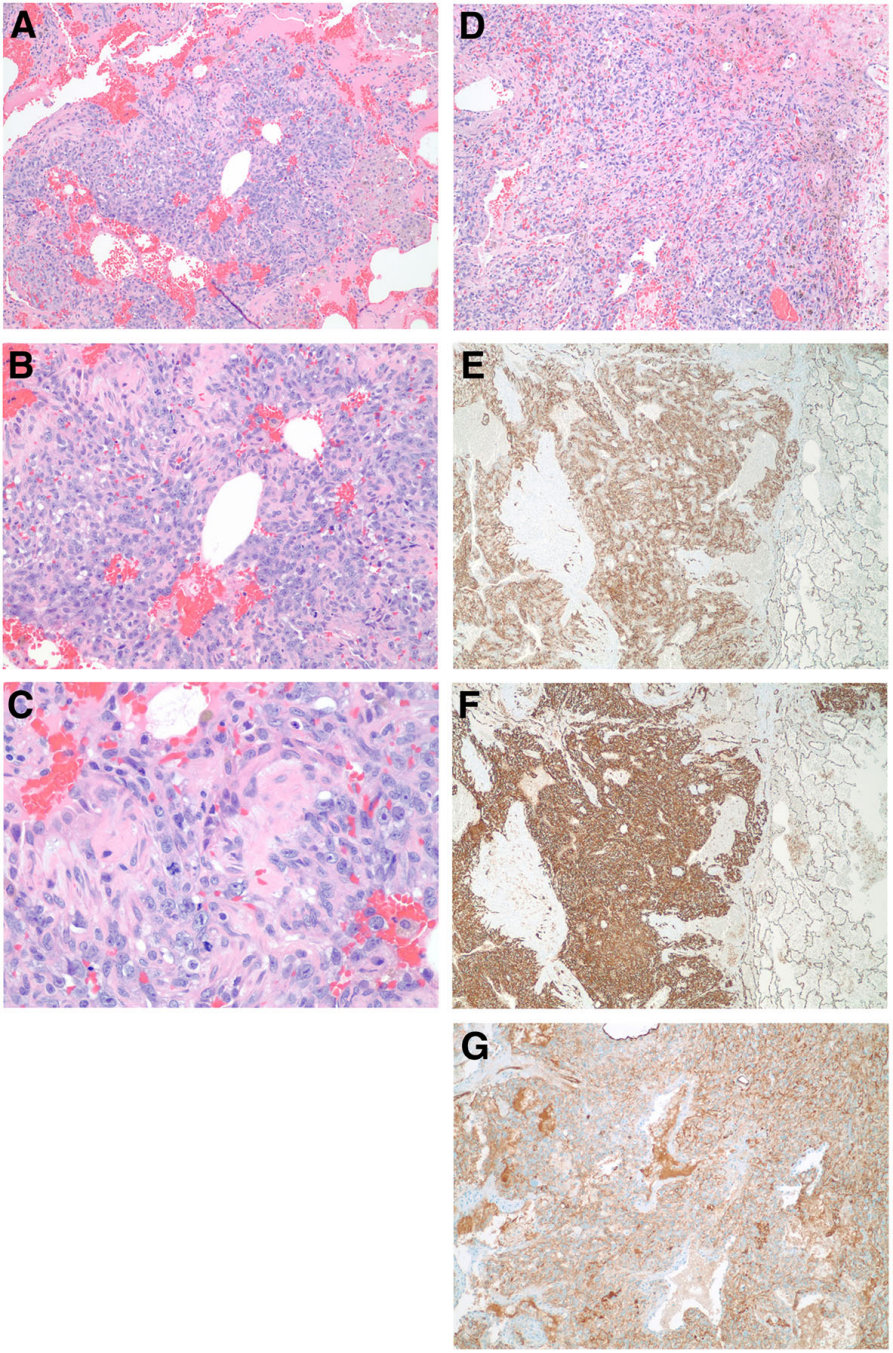
Images of the tumor from the lung. **a**-**d** Hematoxyalin and eosin-stained tumor sections from lung nodules. **a** Low power magnification (40X) of tumor demonstrated nodules of tumor cells can be seen in a background of abundant, fresh blood cells. **b** Medium power magnification (100X) showed the tumor cells are both epitheliod and spindle-shaped in appearance. The epitheliod morphology predominates in this area of the tumor. **c** High power magnification (400X) illustrated the tumor cells have prominent nucleoli. A mitotic figure can be seen in the center of the image confirming the tumor is mitotically active. **d** Another view of the tumor showing both epitheliod and spindle-shaped tumor cells, but in this section the spindle-shaped cells predominant (40X). **e** Tumor cells stained positive for CD34, a vascular marker (40X). **f** Tumor cells showed intense signal for CD31, another vascular marker (40X). **g** Factor VIII-related antigen, another endothelial marker commonly used to identify vascular tumors, demonstrated positivity in the tumor cells (40X)

**Fig. 3:**
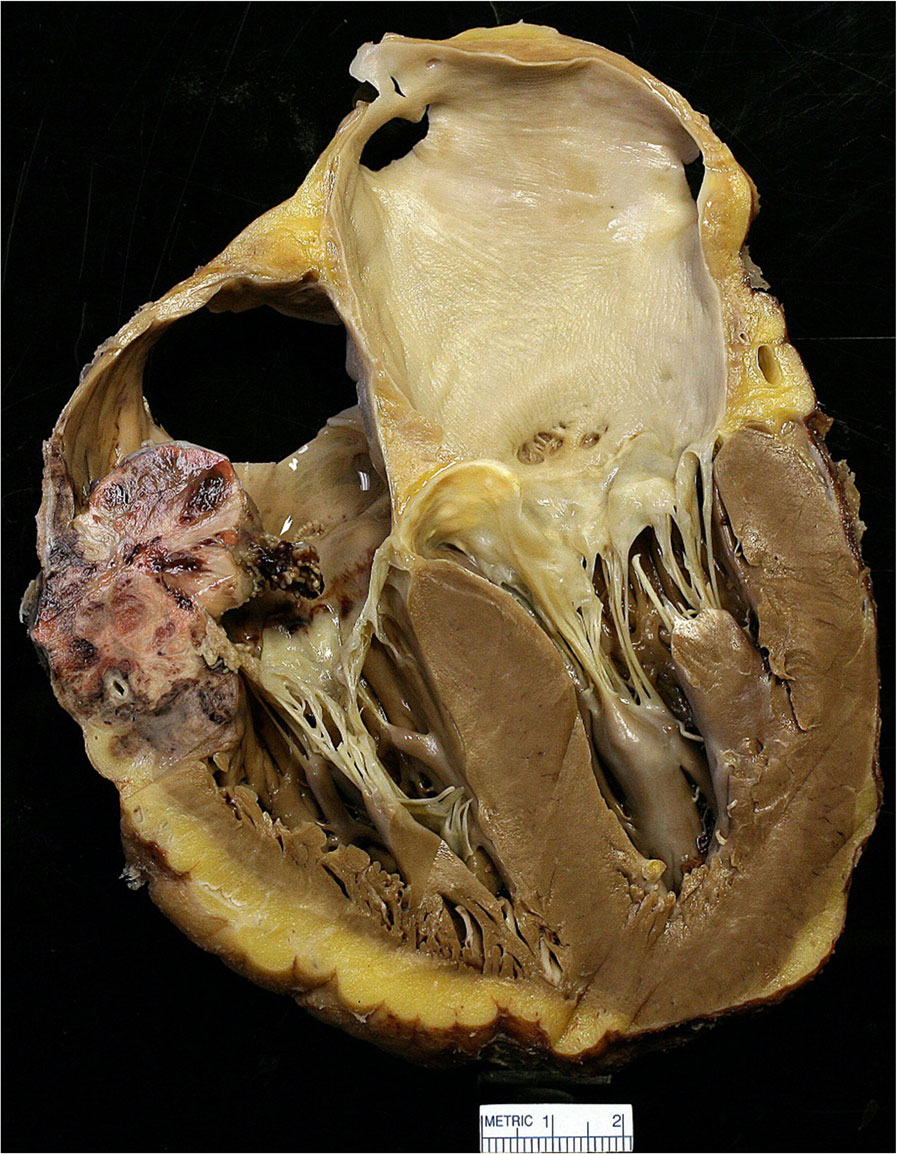
Cardiac angiosarcoma the right atrium. The tumor was approximately 4 cm in diameter and attached just above the tricuspid valve annulus, extending anteriorly and laterally. The tumor extended into the right atrioventricular sulcus and was present throughout the atrioventricular sulcus shortly after the origin of the right coronary artery and extending posteriorly. For more gross images see Podduturi and Guileyardo [32]

This case was presented previously [32]. Briefly, a 56-year-old Caucasian male was transferred to our hospital for possible pericardial window by cardiothoracic surgery after a transthoracic echocardiogram revealed a large pericardial effusion. A computed tomography scan of the chest revealed numerous pulmonary nodules surrounded by ground glass densities, along with small pleural effusions, and moderate to large pericardial effusion. Approximately 1250 ml of fluid was removed by pericardiocentesis, and the fluid was negative for the presence of infection or malignancy. Whole body positron emission tomography scan revealed a large, irregularly shaped, strikingly hypermetabolic cardiac lesion of probable right atrium origin (Fig. 1a). Moreover, there were extensive hypermetabolic pulmonary nodules in a background of ground glass infiltrates (Fig. 1b). A provisional diagnosis of cardiac sarcoma was made, and the patient underwent a left video-assisted thoracotomy, pericardial window and biopsy, and left lower lobe resection. Tumor cells in the lung nodules were both epitheliod and spindle-shaped in appearance. They were immunoreactive for Cluster of Differentiation (CD)31, CD34, and Factor VIII-related antigen, findings that were consistent with an angiosarcoma (Fig. 2). Given the aggressive nature of this cancer, the patient declined treatment and proceeded with in-hospital hospice where he expired on hospital day 19. Postmortem examination showed a large tumor was present in the right atrium attached just cephalad to the tricuspid valve annulus, anteriorly and laterally (Fig. 3). The tumor extended into the right atrioventricular sulcus. The right coronary artery was compressed by the tumor in the right atrioventricular sulcus. The neoplasm extended just slightly into the right ventricular wall but was not present in the ventricular septum, left ventricular free wall, or in the left atrial wall.

## Methods

### Immunohistochemistry

Immunohistochemical staining was performed on tissue sections of formalin-fixed, paraffin-embedded (FFPE) tumor samples using the BenchMarch System (Ventana Medical Systems, Inc., Tucson, AZ). The following primary antibodies were used: An anti-CD31 mouse monoclonal antibody from Dako North America, Inc. (Carpinteria, CA; M0823; lot # 00079267; 1:100 dilution), an anti-CD34 mouse monoclonal antibody from Ventana Medical Systems, Inc (790-2927; lot # 1327304B; 1:1 dilution), and a Factor VIII-related antigen rabbit polyclonal antibody from Cell Marque Corp. (Rocklin, CA; 250A; lot # 1113210A; 1:100 dilution).

### Custom probe design for target enrichment

For this project, we used a set of custom baits (Agilent Technologies, Inc.; Cat# G9496C). Briefly, components of the expanded exome included the following probe groups: original baits from SureSelect Human All Exon V5, (Agilent Technologies, Inc.; Cat # 5190-6209), custom baits for select break point regions which can result in oncogenic fusions (as defined by the COSMIC database v67), and common tumor suppressor transcribed regions (Additional file 1: Table S1). Additionally, to aid in copy number alteration analysis, Agilent 44 k human comparative genomic hybridization (aCGH) probes (Agilent Technologies, Inc.) were supplemented in the regions where there was no strategic bait coverage (Additional file 2: Table S2). Expanded exome resulted in a custom 55.2 Mbp targeted design.

### DNA isolation

Genomic DNA was isolated from frozen blood using the Qiagen QiaAmp DNA Blood Maxi Kit (Qiagen, Inc., Valencia, CA). Specifically, frozen blood (8 ml) was allowed to equilibrate to room temperature and supplemented with 2 ml of PBS. The mixture was then treated with Qiagen protease. Lysis buffer was added to the mixture and DNA isolation was conducted as written per the manufacturer’s the protocol. DNA was eluted in 1000 μl of Buffer AE. The purified DNA was quantified using the Qubit 2.0 Fluorometer (Life Technologies, Grand Island, NY). Absorbance ratios (260/280 and 260/230) were obtained using Nanodrop spectrophotometer (Thermo Fisher Scientific, Inc. Waltham, MA). Tumor DNA was isolated from FFPE tissue using Qiagen AllPrep DNA/RNA FFPE Kit (Qiagen). Briefly, slides with 5–10 μM thick FFPE sections were scraped into microcentrifuge tubes (two total) and treated with 640 μl of Qiagen Deparaffinization Solution. The resulting pellets were dried for 10 min at 37 °C and resuspended in 150 μl of Buffer PKD containing 10 μl proteinase K. After 15 min incubation at 56 °C, samples were cooled on ice for 3 min and then centrifuged at 20,000 x *g* for 15 min. Genomic DNA was isolated from the pellets according to manufacturer’s instructions that included the optional RNAse treatment. Genomic DNA was eluted with 200 μl of buffer ATE and quantified using the Qubit 2.0 Fluorometer (Life Technologies) and Nanodrop spectrophotometer (Thermo Fisher Scientific, Inc.).

### Whole exome library construction and target enrichment

Genomic DNA (500 ng) was sheared in 50 μl of TE low EDTA buffer employing the Covaris E210 system (Covaris, Inc., Woburn, MA) to target fragment sizes of 150–200 bp. Fragmented DNA was then converted to an adapter-ligated whole genome library using the Kapa On-bead Library Prep kit (Kapa Biosciences, Inc., Wilmington, MA; Cat# KK8232) according to the manufacturer’s protocol. SureSelect XT Adaptor Oligo Mix was utilized in the ligation step (Agilent Technologies, Inc.; Cat# 5190-3619). Pre-capture libraries were amplified using SureSelect XT primers (Cat# 5190-3620 and Cat# 5972-3694) for nine cycles. Amplified products were quantified and quality tested using Qubit® dsDNA BR Assay Kit (Life Technologies) and the Bioanalyzer DNA 1000 chip (Agilent Technologies, Inc.). Libraries were then hybridized to a custom Agilent SureSelect bait library (custom content regions are provided in Additional file 1: Table S1). Hybridization reactions were set up with 750 ng of the adapted library according to the SureSelect XT protocol with 24 h incubation at 65 °C followed by post-hybridization washes. SureSelect XT indexes were added to the individual libraries during the eight-cycle post-capture amplification step. Final captures were quantified and quality tested using Qubit® dsDNA HS Assay Kit (Life Technologies) and Bioanalyzer DNA HS chip (Agilent Technologies, Inc).

### Whole exome sequencing and analysis

The sequencing pool was created by evenly combining four uniquely indexed captures into one pool which was sequenced across three lanes on Illumina HiSeq 2500 high output mode at 14 pM clustering density using paired-end reads (Illumina, Inc.). All sequencing reads were converted to industry standard FASTQ files using the Bcl Conversion and Demultiplexing tool (Illumina, Inc). Sequencing reads were aligned to the GRCh37 reference genome using the MEM module of Burrows-Wheeler Aligner (BWA) v0.7.8 [33] and SAMTOOLS v0.1.19 [33] to produce BAM files. After alignment, the base quality scores were recalibrated and joint small insertions and deletions (INDEL) realignment was performed on the BAM files using GATK v3.1-1 [34]. Duplicate read pairs were marked using PICARD v1.111 [35]. Final BAM files were then used to identify germline and somatic events. Germline SNP and INDELS were identified using GATK haplotype caller in the constitutional sample.

Somatic single nucleotide variations (SNVs) and INDELs were identified using SEURAT somatic variant caller [36]. Somatic copy number detection was based on a log2 comparison of normalized physical coverage (or clonal coverage) across tumor and normal whole exome sequencing data, where physical coverage was calculated by considering the entire region a paired-end fragment span. Normal and tumor physical coverage was then normalized, smoothed and filtered for highly repetitive regions prior to calculating the log2 comparison. Loss of Heterozygosity (LOH) is also deduced by calculating alternate allele frequencies for SNPs. Briefly, B-allele frequencies (BAF) are allele fraction of non-reference reads in the tumor at heterzogote common polymorphic SNPs (minor allele frequency >5%) from 1000 genomes [37] in the patient’s germline. Only heterozygous SNPs are plotted determined from that patient’s germline calls. We use the calculation alt/(ref + alt) where alt is B. This should be 50/50 unless loss of heterozygosity (LOH) or allele imbalance has occurred at that site. The BAF can then be plotted against map position to identify regions of LOH. Copy number analysis was also performed using the circular binary segmentation algorithm DNA copy within the BioConductor package [38]. Translocation detection was based on discordant read evidence in the tumor exome sequencing data compared to its corresponding normal data. In order for the structural variant to be called there needs to be greater than 7 read pairs mapping to both sides of the breakpoint [37]. Somatic structural rearrangements were identified through discordant read pairs using a previously published algorithm [39].

### Annotation of somatic data

Somatic events are annotated using SNPeff (http://snpeff.sourceforge.net/), providing basic map position information as well as additional annotation fields for mutations and copy number events. For mutations, we use several functional prediction tools to help prioritize variants that might confer detrimental functional consequences. We use Mutation Assessor [40], Mutation Taster2 [41], and PolyPhen-2 [42]. Mutation Assessor uses a multiple sequence alignment (MSA), partitioned to reflect functional specificity, and generates conservation scores for each column to represent the functional impact of a missense variant [40]. A conservation score is combined with a specificity score to determine a functional impact score. Variants classed as ‘neutral’ or ‘low’ are predicted to not impact protein function, whereas variants classed as ‘medium’ or ‘high’ are predicted to result in altered function. Mutation Taster2 uses Bayesian classification and evolutionary conservation models to determine if a variant is likely neutral or deleterious [41]. PolyPhen-2 uses high-quality multiple protein sequence alignment and machine-learning classification to predict the impact of sequence variants (Benign, Probable Deleterious, or Deleterious) on the stability and function of a protein using structural and comparative evolutionary considerations [42].

## Results

### Somatic alterations in primary cardiac angiosarcoma

We performed next generation sequencing on tumor and germline DNA from a 56-year old male patient with diagnosed primary cardiac angiosarcoma using a 55.2 Mbp custom whole exome capture kit. Sequencing statistics are provided in Additional file 1: Table S1. On-target coverages of 282X for the tumor and 394X for the germline were achieved. Importantly, the tumor sample was FFPE and the blood was fresh. However quality sequencing data was generated from both samples evident by sequencing statistics. Importantly, we generated more sequencing reads from the tumor sample, but fewer of these sequencing reads aligned to the exome target regions as there were likely more low quality reads generated from this FFPE sample (Additional file 1: Table S1). Variant detection algorithms take into account read and base quality to help ensure accuracy in allele determination. This can also have effects on copy number analysis as well. A measure of the noise in log2(tumor/normal) copy number comparison or essentially log2(FFPE/Fresh) is the derivative log2ratio spread or DLR Spread (DLRS). The DLRS statistic describes the absolute value of the log2 ratio variance from each probe to the next, averaged over the entire genome [43]. It is a common measure of noise for CGH arrays [43]. We can also calculate DLRS for copy number from Next Generation Sequencing data using the same algorithm using coverage ratios rather than microarray probe intensities. We can use a similar DLRS threshold as CGH arrays for data of quality enough to make accurate copy number calls, where a DLRS of <0.3 represents low enough noise to make accurate gain and deletion calls. In our hands, copy number from fresh blood normal DNA and fresh *t*umor DNA typically averages DLRS <0.15, indicative of high quality data. The fresh blood sample and FFPE sample used in our study has a DLRS of 0.168 using the copy number tool, supporting high quality copy number analysis for our samples.

**Fig. 4:**
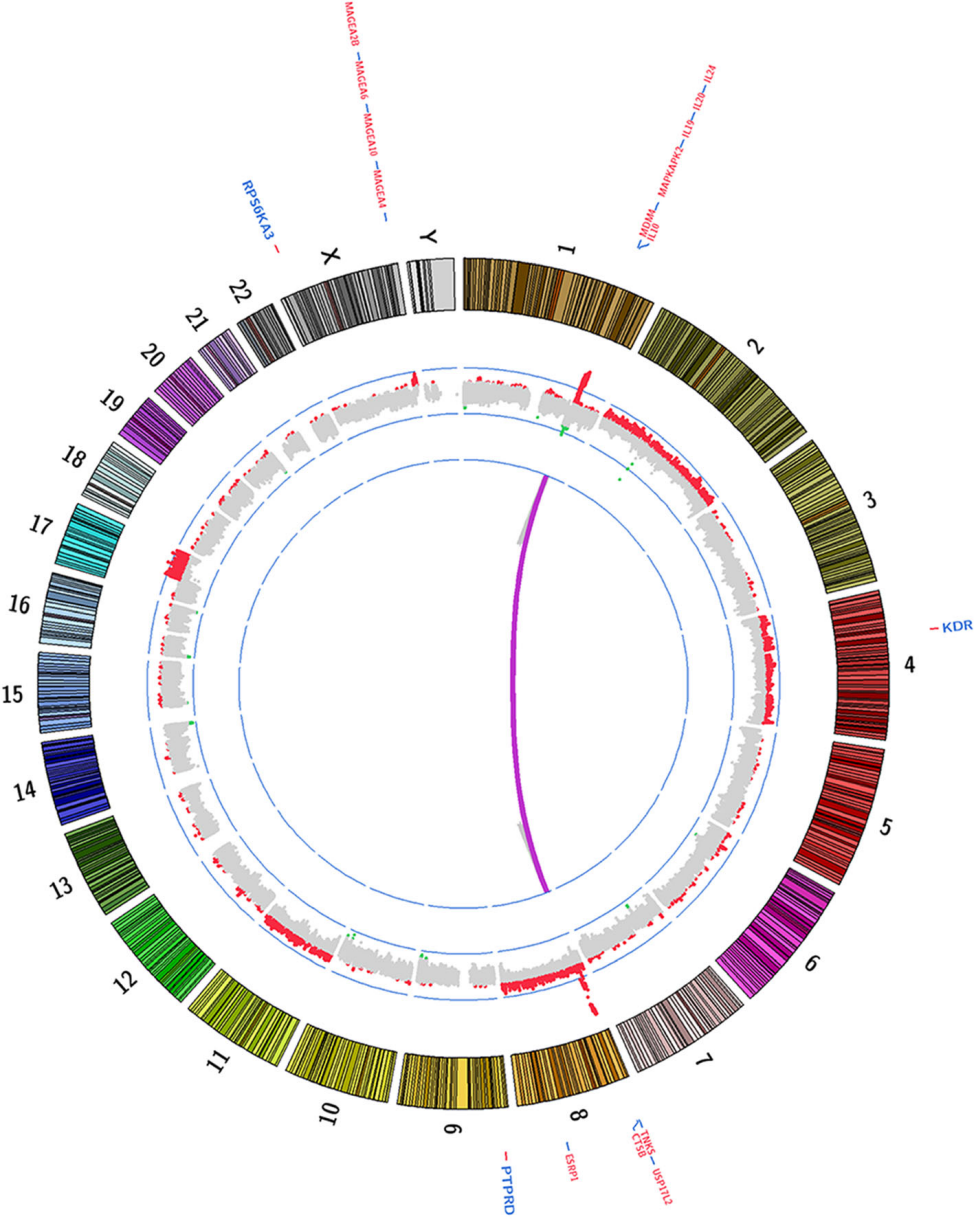
Circos plot with whole exome sequencing summary. Copy number alterations (inside track, *red* = amplifications, *green* = deletions), Select somatic SNVs (outside track, *blue*) and structural events (*purple lines*)

An overview of the tumor genome is provided in the form of a Circos plot (Fig. 4). Using SEURAT variant caller, we identified 57 somatic coding SNVs in this patient’s tumor DNA (Additional file 1: Table S1). Recurrent *PTPRB* and *PLCG1* mutations have been previously reported in this tumor type [31]; however, no somatic coding mutations affecting either gene were found in our data set. Annotations of mutations were provided through SNPeff using several functional prediction algorithms including Mutation Assessor [40], Mutation Taster2 [41], and PolyPhen-2 [42]. Somatic mutations classified as medium or high by Mutation Assessor, Deleterious by MutationTaster2 and Polyphen2 were prioritized (Additional file 1: Table S1). Of note was the presence of a *KDR* mutation at position G681R mapping to the Immunoglobulin (Su(var)3–9, Enhancer-of-zeste, Trithorax (I-SET) protein domain (Fig. 4; Additional file 2: Table S2). Mutations in *KDR* that result in constitutive activation of the Vascular Endothelial Growth Factor Receptor 2 (VEGFR2) receptor leading to induction of angiogenesis signaling have been reported previously for other angiosarcoma tumors [30]. This KDR G681R mutation in the *KDR* gene to be damaging by all prediction algorithms, however the true functional consequences of this mutation are unknown. Another mutation predicted to be damaging by all three prediction algorithms was Cullin 3 (*CUL3*) R247Q (Additional file 1: Table S1). *CUL3* encodes cullin 3, a core component of the multiple cullin-RING-based BCR (BTB- CUL3-RBX1) E3 ubiquitin-protein ligase complexes which mediate the ubiquitination and subsequent proteasomal degradation of target proteins [44]. It is involved in Aurora Kinase and cell cycle control of G1/S checkpoint [45].

Custom probe tiling in our targeted assay allowed for even genome-wide copy number analysis. We detected whole chromosome gains of chromosomes 2, 4, 8 and 11 (Fig. 4). This tumor also displayed a low level broad gain that spanned chromosome (chr)17q12-qter. More focal events were detected at 1q, 8p and Xq (Fig. 4; Additional file 3: Figure S1). A focal gain was observed at chrXq28 that included a number of cancer testis antigens including Melanoma Antigen Family members (*MAGE*) *-A1*, -*A2*, -*A3*, -*A4*, -*A6*, -*A10*, -*A12*, -*2B, CTAG1A* and *CTAG2*. The chr1 event was characterized by an allele shift and several focal regions of amplification spanning an approximate 7 Mb region at 1q32 (Additional file 2: Table S2 and Additional file 4: Figure S2). Regions of amplification at this locus encompassed a number of cancer-related genes including *MDM4,* Mitogen-Activated Protein Kinase-Activated Protein Kinase 2 (*MAPKAPK2*), and Phosphatidylinositol-4-Phosphate 3-Kinase Catalytic Subunit Type 2 Beta (*PIK3C2B*) and several interleukin genes including *IL10*, *IL19*, *IL20*, and *IL24* (Additional file 2: Table S2). The event at chr8p was also characterized by an allele shift and several focal regions of amplification and spanned an approximate 5 M region at 8p23 (Additional file 2: Table S2 and Additional file 5: Figure S3). This region encompassed tankyrase 1 gene, *TNKS*, which acts is an activator of the Wingless-Int (Wnt) signaling pathway [46]. It also contains the GATA Binding Protein 4 (*GATA4*) gene, which has highest expression in heart tissue and promotes cardiac myocyte enlargement [47]. Other significantly amplified genes found at the 8p23 locus included Ubiquitin Specific Peptidase 17-Like Family Member 2 (*USP17L2*), Cathepsin B (*CTSB*), and Epithelial Splicing Regulatory Protein 1 (*ESPR1*) (Additional file 2: Table S2).

## Conclusions

In this report we characterized the genomic landscape of a rare primary cardiac angiosarcoma tumor taken postmortem from a 56 year old male patient. We have identified a number of somatic copy number events, mostly marked by amplifications, and SNV alterations in this patient’s tumor. Our approach did not uncover any translocations that could result in oncogenic fusions previously reported in primary angiosarcomas [48, 49]. Copy number coupled with the translocation analysis suggested a presence of a circular chromosome harboring the *MDM4* gene. We were able to confirm our hypothesis by *in silico* reconstructing the discordant read pairs mapping to multiple regions on chr1 and chr8. Double minute chromosomes and homogeneously stained regions have been identified in several tumor types and are biologically complex extrachromosomal entities frequently harboring known oncogenes (i.e *MYC*) and/or oncogenic fusions [50]. Their origin in the nucleus is quite intriguing and is thought to arise as a result of several mechanisms including chromothripsis, a multi-step product of complex duplications and recombinations through non-homologous end joining. To our knowledge, there are no previous reports describing circular extrachromosomal DNA in primary angiosarcomas or angiosarcomas of the heart carrying *MDM4* gene. Future studies investigating frequency of this occurrence in primary cardiac angiosarcomas could add clinical value.

Another amplified region in this tumor genome mapped to chrXq28. This locus encompasses the *MAGE* gene cluster. Genes belonging to this cluster, cancer testis antigens (CTAs), often exhibit tumor-specific expression and are currently explored as immunotherapeutic targets in sarcomas [51], other solid tumors and hematologic malignancies [52]. Several ongoing preclinical and phase I trials are investigating vaccination-induced immunotherapeutic response in sarcoma patients with advanced disease (NCT02054104, NCT01313429, and NCT013441496).

Interestingly, a recent report describes an association between MAGE-A and MDM2 oncogene, E3 ubiquitin protein ligase (MDM2)/MDM4 complex in vitro in a human cell line model [53]. Specifically, MAGE-A was shown to compete with MDM4 for binding to MDM2 leading to elevated levels of free MDM4. Additionally, the group showed a positive correlation between MAGE-A and MDM4 by immunohistochemistry in primary breast cancer. Although we cannot comment on expression or protein levels of either MDM4 or MAGE in this patient’s tumor, the co-amplification significance of multiple MAGE genes and MDM4 may merit further exploration in this tumor type.

Recently, another patient presented to our clinic with a cardiac angiosarcoma and a Foundation One genetic profile was performed on his tumor. A mutation of unknown significance in *KDR* was observed (N704del) in a region where other mutations, deletions, and insertions have been reported [54], including the patient described in the present study. Additionally, amplification of *MDM4* was found as was seen in our initial patient. Thus, from a potential therapeutic viewpoint one could one take away several points from these whole exome sequencing results (fully realizing this is from only two patients). Firstly, the *KDR*-G681R mutation, which occurs within the second I-SET domain of this protein, is novel. The G681 amino acid is highly conserved throughout evolution suggesting that it is a functionally relevant residue and based on prediction algorithms is potentially functionally relevant, however this would need to be validated through in vitro and/or in vivo cell-based mechanistic approaches. Although the true functional relevance of the mutation is currently unknown, this mutation would confer the strongest therapeutic targeting potential. One could consider a vascular endothelial growth factor receptor directed therapy such as bevacizumab, pazopanib, cabozantinib, vandetanib, ziv-aflibercept, or ramucirumab. Of note is that sorafenib and sunitnib inihibited KDR activity in vitro as previously noted by Antonescu and colleagues [30]. Secondly, there was high level focal amplification at 1q32, which contains the *MDM4* gene. Although there are multiple anticancer agents under clinical development which inhibit the structurally similar p53 binding protein MDM2 (e.g., the Nutlins etc.) there have not been any under development which inhibits MDM4 or MDMX (any MDM protein). But that has changed with the ongoing development of the agent ALRN-6924 an MDM2/MDMX dual inhibitor (which reactivates p53) [55]. That agent would be a potential match against the amplified MDM4 target. Although we have provided a relatively high resolution view of the genomic landscape of this tumor, further studies are needed to determine the effect of candidate cancer genes hypothesized from our analysis, and clinical validation of the frequency of these events in cardiac angiosarcomas.

## Additional files

Additional file 1: Table S1. List of coordinates and genomic regions submitted to Sure Design software for custom bait selection. (DOCX 18 kb)
Additional file 2: Table S2. Basic Next Generation Sequencing Whole Exome Alignment Statistics. (DOCX 13 kb)
Additional file 3: Figure S1. Genome-wide chromosomal copy number plot outputs from 1A) copy number analysis, 1B) LOH and allelic imbalance analysis, 1C) segmentation algorithm copy number analysis, and 1D) ExomeCNV copy number analysis. 1A) chromosomal plots contain chromosomal map position in megabases on the X-axis, and the log2 fold change ratio information on the Y-axis. Regions of copy neutrality (ratios between log2FC -0.75 and +0.75) are black, regions of copy number gain (ratio > log2FC 0.75) are red, and regions of copy number loss (ratios < log2FC -0.75) are green. 1B) chromosomal plots contain chromosomal map position in megabases on the X-axis, and the B-allele frequency (BAF) on the Y-axis revealing chromosomal allelic imbalances. 1C) the copy number log2 fold change ratios (Y-axis) for each chromosome are colored alternately in green and black across the entirety of the genome map positions along the X-axis. 1D) chromosomal plots contain chromosomal map position in megabases on the X-axis, and the log2 fold change ratio information on the Y-axis derived from ExomeCNV. (ZIP 7726 kb)
Additional file 4: Figure S2. Chromosome 1 copy number plot outputs illustrating 1q32 amplification. Plots represent outputs from (2A upper panel) LOH and allelic imbalance analysis, and (2A lower panel) copy number analysis. 2A upper panel, chromosome 1 plot with map position in megabases on the X-axis, and the B-allele frequency (BAF) on the Y-axis revealing chromosomal allelic imbalances. 2A lower panel, chromosome 1 plot with map position in megabases on the X-axis, and the log2 fold change ratio information on the Y-axis. 2B shows 10 megabase zoomed region of chromosome 1 (1q32) amplicon. 2B upper panel, chromosome 1 zoomed in plot with map position in megabases on the X-axis, and the B-allele frequency (BAF) on the Y-axis revealing chromosomal allelic imbalance at this region. 2B lower panel, chromosome 1 zoomed in plot with map position in megabases on the X-axis, and the log2 fold change ratio information on the Y-axis. The map postion related to the *MDM4* gene locus is depicted as a purple line on each plot. (ZIP 1288 kb)
Additional file 5: Figure S3. Chromosome 8 copy number plot outputs illustrating 8p allelic imbalance and 8p23 amplification. Plots represent outputs from (upper panel) custom algorithm providing information on allelic imbalances, and (lower panel) copy number analysis. Upper panel, chromosome 8 plot with map position in megabases on the X-axis, and the B-allele frequency (BAF) on the Y-axis revealing chromosomal allelic imbalances. Lower panel, chromosome 8 plot with map position in megabases on the X-axis, and the log2 fold change ratio information on the Y-axis. (JPG 1099 kb)

## Abbreviations

aCGH: array Comparative Genomic Hybridization
BAF: B-allele frequencies
BWA: Burrows-Wheeler Aligner
CD: Cluster of differentiation
CDKN2A: Cyclin Dependent Kinase Inhibitor 2A
chr: chromosome
CNS: Central nervous system
CTAG1A: Cancer/Testis Antigen 1A
CTAG2: Cancer/Testis Antigen 2
CTAs: Cancer testis antigens
CTSB: Cathepsin B
CUL3: Cullin 3
DLRS: Derivative log2ratio spread
ESPR1: Epithelial Splicing Regulatory Protein 1
FFPE: Formalin-Fixed, Paraffin-Embedded
FLT1: Fms Related Tyrosine Kinase 1
FLT4: Fms Related Tyrosine Kinase 4
GATA4: GATA Binding Protein 4
IL: Interleukin
INDELs: Insertions and deletions
I-SET domain: Immunoglobulin (Su(var)3-9, Enhancer-of-zeste, Trithorax)
KDR: Kinase Insert Domain Receptor
KRAS: V-Ki-ras2 Kirsten rat sarcoma viral oncogene homolog
LOH: Loss of heterozygosity
MAGE-A1 – 2B: Melanoma Antigen Family Member A1 – 2B
MAPKAPK2: Mitogen-Activated Protein Kinase-Activated Protein Kinase 2
MDM2: MDM2 oncogene, E3 ubiquitin protein ligase
MDM4: MDM4 p53 binding protein homolog
MDMX: Any MDM protein
MSA: Multiple sequence alignment
MYC: V-Myc Avian Myelocytomatosis Viral Oncogene Homolog
PIK3C2B: Phosphatidylinositol-4-Phosphate 3-Kinase Catalytic Subunit Type 2 Beta
PLCG1: Phospholipase C, gamma 1
PTPRB: Protein tyrosine phosphatase, receptor type, B
SNRK: SNF Related Kinase
SNVs: Single nucleotide variations
TEK: TEK Receptor Tyrosine Kinase
TIE1: Tyrosine Kinase with Immunoglobulin like and EGF Like Domains 1
TNKS: Tankyrase 1
TP53: tumor protein P53
USP17L2: Ubiquitin Specific Peptidase 17-Like Family Member 2
VEGFR2: Vascular endothelial growth factor receptor 2
Wnt: Wingless-Int

## References

[1] Lam KY, Dickens P, Chan AC (1993). Tumors of the heart. A 20-year experience with a review of 12,485 consecutive autopsies. Arch Pathol Lab Med.

[2] Reynen K (1996). Frequency of primary tumors of the heart. Am J Cardiol.

[3] Burke A, Virmani R (1996). More on cardiac myxomas. N Engl J Med.

[4] Janigan DT, Husain A, Robinson NA (1986). Cardiac angiosarcomas. A review and a case report. Cancer.

[5] Gal AA, Koss MN, McCarthy WF, Hochholzer L (1994). Prognostic factors in pulmonary fibrohistiocytic lesions. Cancer.

[6] Burke AP, Tazelaar H, Butany JW, El-Demellawy D, Loire R, Geva T, Galvin JR, Veinot JP, Virmani R, Kamiya H, William TD, Brambilla E, Muller-Hermelink HK, Harris CC (2004). Cardiac Sarcomas. Pathology and genetics tumours of the lung, pleura, thymus and heart.

[7] Majano-Lainez RA (1997). Cardiac tumors: a current clinical and pathological perspective. Crit Rev Oncog.

[8] Meng Q, Lai H, Lima J, Tong W, Qian Y, Lai S (2002). Echocardiographic and pathologic characteristics of primary cardiac tumors: a study of 149 cases. Int J Cardiol.

[9] Glancy DL, Morales JB, Roberts WC (1968). Angiosarcoma of the heart. Am J Cardiol.

[10] Roberts WC (2001). Neoplasms involving the heart, their simulators, and adverse consequences of their therapy. Proc (Bayl Univ Med Cent).

[11] Fernandes F, Soufen HN, Ianni BM, Arteaga E, Ramires FJ, Mady C (2001). Primary neoplasms of the heart. Clinical and histological presentation of 50 cases. Arq Bras Cardiol.

[12] Jimenez Mazuecos JM, Fuentes Manso R, Segovia Cubero J, Toquero Ramos J, Oteo Dominguez JF, Alonso-Pulpon Rivera L (2003). Is heart transplantation for primary cardiac sarcoma a useful therapeutic option?. Rev Esp Cardiol.

[13] Crespo MG, Pulpon LA, Pradas G, Serrano S, Segovia J, Vegazo I, Salas C, Espana P, Silva L, Burgos R (1993). Heart transplantation for cardiac angiosarcoma: should its indication be questioned?. J Heart Lung Transplant.

[14] Talbot SM, Taub RN, Keohan ML, Edwards N, Galantowicz ME, Schulman LL (2002). Combined heart and lung transplantation for unresectable primary cardiac sarcoma. J Thorac Cardiovasc Surg.

[15] Orlandi A, Ferlosio A, Roselli M, Chiariello L, Spagnoli LG (2010). Cardiac sarcomas: an update. J Thorac Oncol.

[16] Ge Y, Ro JY, Kim D, Kim CH, Reardon MJ, Blackmon S, Zhai J, Coffey D, Benjamin RS, Ayala AG (2011). Clinicopathologic and immunohistochemical characteristics of adult primary cardiac angiosarcomas: analysis of 10 cases. Ann Diagn Pathol.

[17] Penel N, Marreaud S, Robin YM, Hohenberger P (2011). Angiosarcoma: state of the art and perspectives. Crit Rev Oncol Hematol.

[18] Maki RG, D’Adamo DR, Keohan ML, Saulle M, Schuetze SM, Undevia SD, Livingston MB, Cooney MM, Hensley ML, Mita MM (2009). Phase II study of sorafenib in patients with metastatic or recurrent sarcomas. J Clin Oncol.

[19] Chugh R, Wathen JK, Maki RG, Benjamin RS, Patel SR, Meyers PA, Priebat DA, Reinke DK, Thomas DG, Keohan ML (2009). Phase II multicenter trial of imatinib in 10 histologic subtypes of sarcoma using a bayesian hierarchical statistical model. J Clin Oncol.

[20] Agulnik M, Yarber JL, Okuno SH, von Mehren M, Jovanovic BD, Brockstein BE, Evens AM, Benjamin RS (2013). An open-label, multicenter, phase II study of bevacizumab for the treatment of angiosarcoma and epithelioid hemangioendotheliomas. Ann Oncol.

[21] Manner J, Radlwimmer B, Hohenberger P, Mossinger K, Kuffer S, Sauer C, Belharazem D, Zettl A, Coindre JM, Hallermann C (2010). MYC high level gene amplification is a distinctive feature of angiosarcomas after irradiation or chronic lymphedema. Am J Pathol.

[22] Guo T, Zhang L, Chang NE, Singer S, Maki RG, Antonescu CR (2011). Consistent MYC and FLT4 gene amplification in radiation-induced angiosarcoma but not in other radiation-associated atypical vascular lesions. Genes Chromosomes Cancer.

[23] Przygodzki RM, Finkelstein SD, Keohavong P, Zhu D, Bakker A, Swalsky PA, Soini Y, Ishak KG, Bennett WP (1997). Sporadic and Thorotrast-induced angiosarcomas of the liver manifest frequent and multiple point mutations in K-ras-2. Lab Invest.

[24] Chang T-G, Wang J, Chen L-W, Chang H-W, Chen J-S, Cho C-L (1997). Loss of expression of the p16 gene is frequent in malignant skin tumors. Biochem Biophys Res Commun.

[25] Tannapfel A, Weihrauch M, Benicke M, Uhlmann D, Hauss J, Wrbitzky R, Wittekind C (2001). p16INK4A-alterations in primary angiosarcoma of the liver. J Hepatol.

[26] Weihrauch M, Markwarth A, Lehnert G, Wittekind C, Wrbitzky R, Tannapfel A (2002). Abnormalities of the ARF-p53 pathway in primary angiosarcomas of the liver. Hum Pathol.

[27] Naka N, Tomita Y, Nakanishi H, Araki N, Hongyo T, Ochi T, Aozasa K (1997). Mutations of p53 tumor-suppressor gene in angiosarcoma. Int J Cancer.

[28] Zu Y, Perle MA, Yan Z, Liu J, Kumar A, Waisman J (2001). Chromosomal abnormalities and p53 gene mutation in a cardiac angiosarcoma. Appl Immunohistochem Mol Morphol.

[29] Hayashi T, Koike K, Kumasaka T, Saito T, Mitani K, Terao Y, Ogishima D, Yao T, Takeda S, Takahashi K (2012). Uterine angiosarcoma associated with lymphangioleiomyomatosis in a patient with tuberous sclerosis complex: an autopsy case report with immunohistochemical and genetic analysis. Hum Pathol.

[30] Antonescu CR, Yoshida A, Guo T, Chang NE, Zhang L, Agaram NP, Qin LX, Brennan MF, Singer S, Maki RG (2009). KDR activating mutations in human angiosarcomas are sensitive to specific kinase inhibitors. Cancer Res.

[31] Behjati S, Tarpey PS, Sheldon H, Martincorena I, Van Loo P, Gundem G, Wedge DC, Ramakrishna M, Cooke SL, Pillay N (2014). Recurrent PTPRB and PLCG1 mutations in angiosarcoma. Nat Genet.

[32] Podduturi V, Guileyardo JM (2014). Primary cardiac angiosarcoma. Acad Forensic Pathol.

[33] Li H, Handsaker B, Wysoker A, Fennell T, Ruan J, Homer N, Marth G, Abecasis G, Durbin R, Genome Project Data Processing S (2009). The Sequence Alignment/Map format and SAMtools. Bioinformatics.

[34] McKenna A, Hanna M, Banks E, Sivachenko A, Cibulskis K, Kernytsky A, Garimella K, Altshuler D, Gabriel S, Daly M (2010). The Genome Analysis Toolkit: a MapReduce framework for analyzing next-generation DNA sequencing data. Genome Res.

[35] PICARD v1.111. http://broadinstitute.github.io/picard/.

[36] Christoforides A, Carpten JD, Weiss GJ, Demeure MJ, Von Hoff DD, Craig DW (2013). Identification of somatic mutations in cancer through Bayesian-based analysis of sequenced genome pairs. BMC Genomics.

[37] Genomes Project C, Auton A, Brooks LD, Durbin RM, Garrison EP, Kang HM, Korbel JO, Marchini JL, McCarthy S, McVean GA (2015). A global reference for human genetic variation. Nature.

[38] Seshan VE, Olshen AR. DNAcopy: DNA copy number data analysis. 2016. In: R package version 1.44.0.

[39] Liang WS, Aldrich J, Tembe W, Kurdoglu A, Cherni I, Phillips L, Reiman R, Baker A, Weiss GJ, Carpten JD (2014). Long insert whole genome sequencing for copy number variant and translocation detection. Nucleic Acids Res.

[40] Reva B, Antipin Y, Sander C (2011). Predicting the functional impact of protein mutations: application to cancer genomics. Nucleic Acids Res.

[41] Schwarz JM, Rodelsperger C, Schuelke M, Seelow D (2010). MutationTaster evaluates disease-causing potential of sequence alterations. Nat Methods.

[42] Adzhubei I, Jordan DM, Sunyaev SR (2013). Predicting functional effect of human missense mutations using PolyPhen-2. Curr Protoc Hum Genet.

[43] Pinto D, Darvishi K, Shi X, Rajan D, Rigler D, Fitzgerald T, Lionel AC, Thiruvahindrapuram B, Macdonald JR, Mills R (2011). Comprehensive assessment of array-based platforms and calling algorithms for detection of copy number variants. Nat Biotechnol.

[44] Zhang HF, Tomida A, Koshimizu R, Ogiso Y, Lei S, Tsuruo T (2004). Cullin 3 promotes proteasomal degradation of the topoisomerase I-DNA covalent complex. Cancer Res.

[45] Sumara I, Peter M (2007). A Cul3-based E3 ligase regulates mitosis and is required to maintain the spindle assembly checkpoint in human cells. Cell Cycle.

[46] Seimiya H, Smith S (2002). The telomeric poly(ADP-ribose) polymerase, tankyrase 1, contains multiple binding sites for telomeric repeat binding factor 1 (TRF1) and a novel acceptor, 182-kDa tankyrase-binding protein (TAB182). J Biol Chem.

[47] Liang Q, De Windt LJ, Witt SA, Kimball TR, Markham BE, Molkentin JD (2001). The transcription factors GATA4 and GATA6 regulate cardiomyocyte hypertrophy in vitro and in vivo. J Biol Chem.

[48] Dunlap JB, Magenis RE, Davis C, Himoe E, Mansoor A (2009). Cytogenetic analysis of a primary bone angiosarcoma. Cancer Genet Cytogenet.

[49] Gru AA, Becker N, Pfeifer JD (2013). Angiosarcoma of the parotid gland with a t(12;22) translocation creating a EWSR1-ATF1 fusion: a diagnostic dilemma. J Clin Pathol.

[50] L’Abbate A, Macchia G, D’Addabbo P, Lonoce A, Tolomeo D, Trombetta D, Kok K, Bartenhagen C, Whelan CW, Palumbo O (2014). Genomic organization and evolution of double minutes/homogeneously staining regions with MYC amplification in human cancer. Nucleic Acids Res.

[51] Wilky BA, Goldberg JM (2014). Immunotherapy in sarcoma: a new frontier. Discov Med.

[52] Bodey B (2002). Cancer-testis antigens: promising targets for antigen directed antineoplastic immunotherapy. Expert Opin Biol Ther.

[53] Marcar L, Ihrig B, Hourihan J, Bray SE, Quinlan PR, Jordan LB, Thompson AM, Hupp TR, Meek DW (2015). MAGE-A Cancer/Testis Antigens Inhibit MDM2 Ubiquitylation Function and Promote Increased Levels of MDM4. PLoS One.

[54] Forbes SA, Beare D, Gunasekaran P, Leung K, Bindal N, Boutselakis H, Ding M, Bamford S, Cole C, Ward S (2015). COSMIC: exploring the world’s knowledge of somatic mutations in human cancer. Nucleic Acids Res.

[55] Chang YS, Graves B, Guerlavais V, Tovar C, Packman K, To KH, Olson KA, Kesavan K, Gangurde P, Mukherjee A (2013). Stapled alpha-helical peptide drug development: a potent dual inhibitor of MDM2 and MDMX for p53-dependent cancer therapy. Proc Natl Acad Sci U S A.

