# Supplementary material for: Case report: whole exome sequencing of primary cardiac angiosarcoma highlights potential for targeted therapies
Source: BMC Cancer. 2017 Jan 5;17:17. doi: 10.1186/s12885-016-3000-z (PMC5217318; doi:10.1186/s12885-016-3000-z)
Supplement: Additional file 1: Table S1. — List of coordinates and genomic regions submitted to Sure Design software for custom bait selection. (DOCX 18 kb) [file 12885_2016_3000_MOESM1_ESM.docx]

**Additional file 1: Table S1**

| **Gene** | | **Chromosome** | **Region start coordinate** | | **Region end coordinate** | | | |
| --- | --- | --- | --- | --- | --- | --- | --- | --- |
| **NTRK1** | | 1 | 156844801 | | 156845311 | | | |
| **NTRK1** | | 1 | 156843752 | | 156844362 | | | |
| **ALK** | | 2 | 29448432 | | 29449787 | | | |
| **ALK** | | 2 | 29446395 | | 29448326 | | | |
| **PDGFRA** | | 4 | 55138688 | | 55139897 | | | |
| **PDGFRA** | | 4 | 55133909 | | 55136799 | | | |
| **PDGFRA** | | 4 | 55139898 | | 55140697 | | | |
| **PDGFRA** | | 4 | 55140793 | | 55141140 | | | |
| **ROS1** | | 6 | 117658334 | | 117650610 | | | |
| **ROS1** | | 6 | 117647386 | | 117645579 | | | |
| **ROS1** | | 6 | 117645494 | | 117642558 | | | |
| **ROS1** | | 6 | 117642421 | | 117641194 | | | |
| **BRAF** | | 7 | 140500161 | | 140494268 | | | |
| **BRAF** | | 7 | 140494107 | | 140487385 | | | |
| **BRAF** | | 7 | 140487347 | | 140482958 | | | |
| **BRAF** | | 7 | 140482820 | | 140481494 | | | |
| **JAK2** | | 9 | 5044521 | | 5050685 | | | |
| **JAK2** | | 9 | 5055789 | | 5064882 | | | |
| **JAK2** | | 9 | 5066790 | | 5069021 | | | |
| **JAK2** | | 9 | 5069209 | | 5069924 | | | |
| **JAK2** | | 9 | 5077581 | | 5078305 | | | |
| **JAK2** | | 9 | 5078445 | | 5080228 | | | |
| **JAK2** | | 9 | 5080684 | | 5081724 | | | |
| **RET** | | 10 | 43604679 | | 43606654 | | | |
| **RET** | | 10 | 43606914 | | 43607546 | | | |
| **RET** | | 10 | 43609124 | | 43609927 | | | |
| **RET** | | 10 | 43610185 | | 43612031 | | | |
| **MAML2** | | 11 | 95825055 | | 95724888 | | | |
| **RARA** | | 17 | 38487649 | | 38504567 | | | |
| **ERG** | | 21 | 40033581 | | 39956870 | | | |
| **ERG** | | 21 | 39956767 | | 39947672 | | | |
| **ERG** | | 21 | 39947585 | | 39817545 | | | |
| **ERG** | | 21 | 39817326 | | 39795484 | | | |
| **ERG** | | 21 | 39795331 | | 39775632 | | | |
| **ERG** | | 21 | 39775427 | | 39774560 | | | |
| **ERG** | | 21 | 39774478 | | 39772568 | | | |
| **ERG** | | 21 | 39774478 | | 39764367 | | | |
| **ERG** | | 21 | 39764297 | | 39763638 | | | |
| **ERG** | | 21 | 39763580 | | 39762965 | | | |
| **ERG** | | 21 | 39762916 | | 39755846 | | | |
| **EWSR1** | | 22 | 29683124 | | 29684594 | | | |
| **EWSR1** | | 22 | 29684776 | | 29687550 | | | |
| **EWSR1** | | 22 | 29687589 | | 29688125 | | | |
| **EWSR1** | | 22 | 29688159 | | 29688476 | | | |
| **PDGFB** | | 22 | 39639905 | | 39631880 | | | |
| **BCR** | | 22 | 23524427 | | 23595985 | | | |
| **BCR** | | 22 | 23631809 | | 23632525 | | | |
| **TFE3** | | X | 48895721 | | 48895640 | | | |
| **TFE3** | | X | 48895534 | | 48891767 | | | |
| **TFE3** | | X | 48896631 | | 48895968 | | | |
|  | |  |  | |  | |  |  |
| **CDKN2A** | Entire transcribed region | | |  | |  | |  |
| **EGFR** | Entire transcribed region | | |  | |  | |  |
| **FGFR1** | Entire transcribed region | | |  | |  | |  |
| **FGFR2** | Entire transcribed region | | |  | |  | |  |
| **FGFR3** | Entire transcribed region | | |  | |  | |  |
| **NF1** | Entire transcribed region | | |  | |  | |  |
| **PTEN** | Entire transcribed region | | |  | |  | |  |
| **PTPRD** | Entire transcribed region | | |  |  |  |  |  |
| **RB1** | Entire transcribed region | | |  |  |  |  |  |
| **TP53** | Entire transcribed region | | |  |  |  |  |  |
| **TRAF3** | Entire transcribed region | | |  |  |  |  |  |
| **TSC1** | Entire transcribed region | | |  |  |  |  |  |
| **TSC2** | Entire transcribed region | | |  |  |  |  |  |
