# Supplementary material for: Case report: whole exome sequencing of primary cardiac angiosarcoma highlights potential for targeted therapies
Source: BMC Cancer. 2017 Jan 5;17:17. doi: 10.1186/s12885-016-3000-z (PMC5217318; doi:10.1186/s12885-016-3000-z)
Supplement: Additional file 2: Table S2. — Basic Next Generation Sequencing Whole Exome Alignment Statistics. (DOCX 13 kb) [file 12885_2016_3000_MOESM2_ESM.docx]

**Additional file 2: Table S2**

| **Sequencing Statistics** |  |
| --- | --- |
| **Tumor** |  |
| Aligned Reads | 710,356,961 |
| Average Target Coverage | 283 |
| % Target Bases at 10X | 98% |
| % Target Bases at 20X | 98% |
| % Target Bases at 30X | 98% |
| % Target Bases at 40X | 97% |
| % Target Bases at 50X | 97% |
| % Target Bases at 100X | 92% |
|  |  |
| **Normal** |  |
| Aligned Reads | 637,884,662 |
| Average Target Coverage | 394 |
| % Target Bases at 10X | 99% |
| % Target Bases at 20X | 98% |
| % Target Bases at 30X | 98% |
| % Target Bases at 40X | 98% |
| % Target Bases at 50X | 98% |
| % Target Bases at 100X | 95% |
