# Supplementary figures and images for: Case report: whole exome sequencing of primary cardiac angiosarcoma highlights potential for targeted therapies
Source: BMC Cancer. 2017 Jan 5;17:17. doi: 10.1186/s12885-016-3000-z (PMC5217318; doi:10.1186/s12885-016-3000-z)

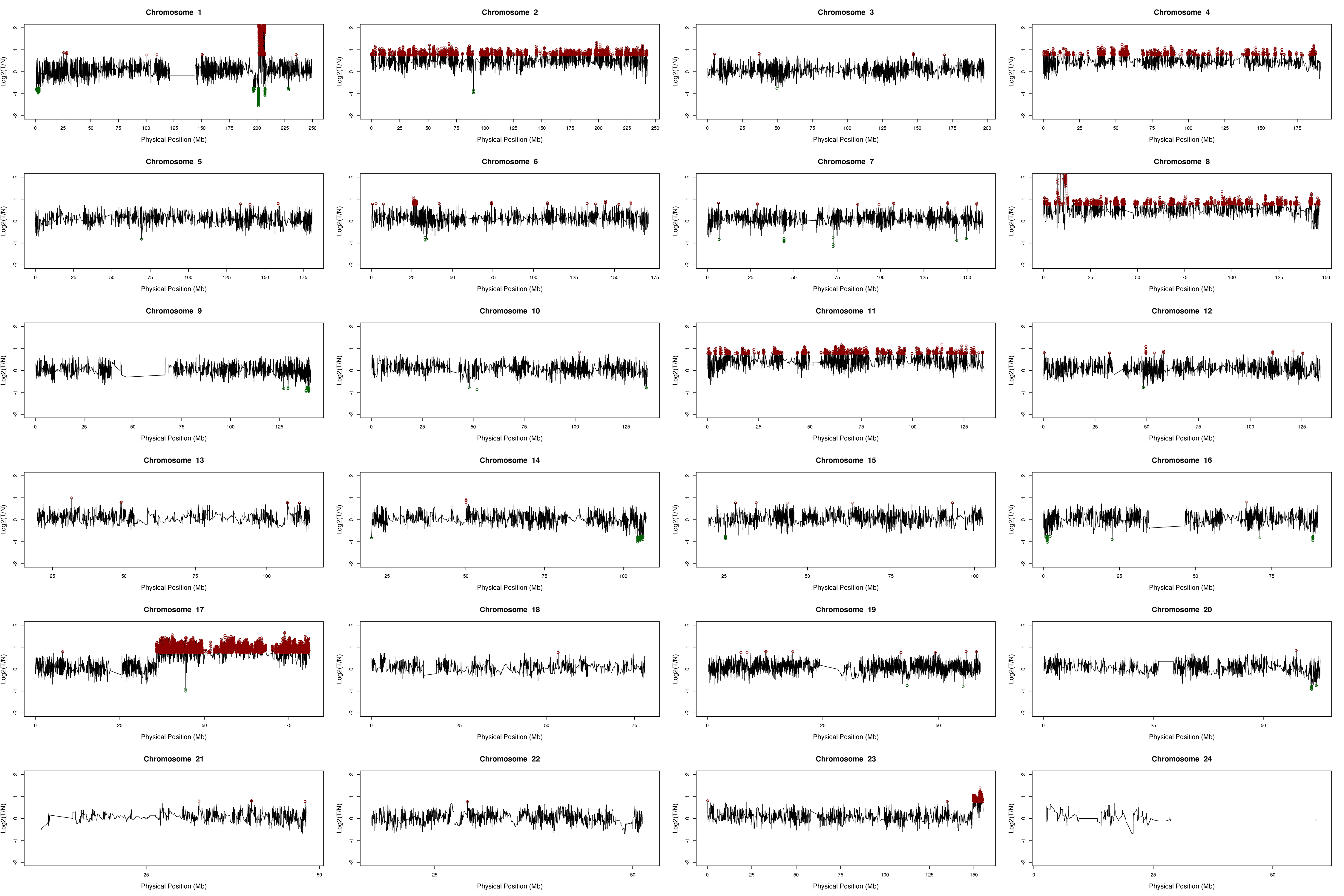

Supplement: Additional file 3: Figure S1. — Genome-wide chromosomal copy number plot outputs from 1A) copy number analysis, 1B) LOH and allelic imbalance analysis, 1C) segmentation algorithm copy number analysis, and 1D) ExomeCNV copy number analysis. 1A) chromosomal plots contain chromosomal map position in megabases on the X-axis, and the log2 fold change ratio information on the Y-axis. Regions of copy neutrality (ratios between log2FC -0.75 and +0.75) are black, regions of copy number gain (ratio > log2FC 0.75) are red, and regions of copy number loss (ratios < log2FC -0.75) are green. 1B) chromosomal plots contain chromosomal map position in megabases on the X-axis, and the B-allele frequency (BAF) on the Y-axis revealing chromosomal allelic imbalances. 1C) the copy number log2 fold change ratios (Y-axis) for each chromosome are colored alternately in green and black across the entirety of the genome map positions along the X-axis. 1D) chromosomal plots contain chromosomal map position in megabases on the X-axis, and the log2 fold change ratio information on the Y-axis derived from ExomeCNV. (ZIP 7726 kb) [file 12885_2016_3000_MOESM3_ESM.zip › Supplementary Figure 1AR4.jpg]

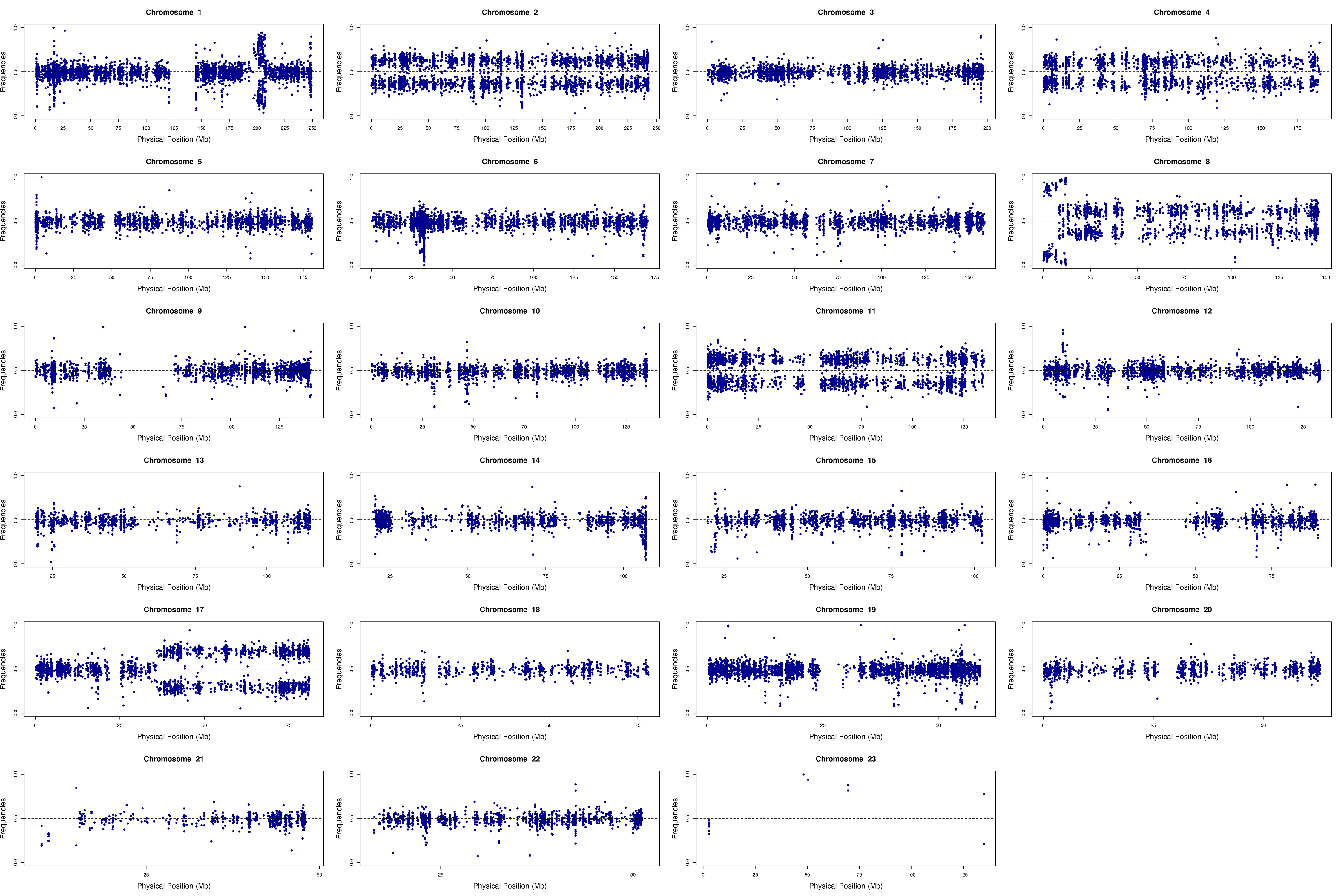

Supplement: Additional file 3: Figure S1. — Genome-wide chromosomal copy number plot outputs from 1A) copy number analysis, 1B) LOH and allelic imbalance analysis, 1C) segmentation algorithm copy number analysis, and 1D) ExomeCNV copy number analysis. 1A) chromosomal plots contain chromosomal map position in megabases on the X-axis, and the log2 fold change ratio information on the Y-axis. Regions of copy neutrality (ratios between log2FC -0.75 and +0.75) are black, regions of copy number gain (ratio > log2FC 0.75) are red, and regions of copy number loss (ratios < log2FC -0.75) are green. 1B) chromosomal plots contain chromosomal map position in megabases on the X-axis, and the B-allele frequency (BAF) on the Y-axis revealing chromosomal allelic imbalances. 1C) the copy number log2 fold change ratios (Y-axis) for each chromosome are colored alternately in green and black across the entirety of the genome map positions along the X-axis. 1D) chromosomal plots contain chromosomal map position in megabases on the X-axis, and the log2 fold change ratio information on the Y-axis derived from ExomeCNV. (ZIP 7726 kb) [file 12885_2016_3000_MOESM3_ESM.zip › Supplementary Figure 1BR4.jpg]

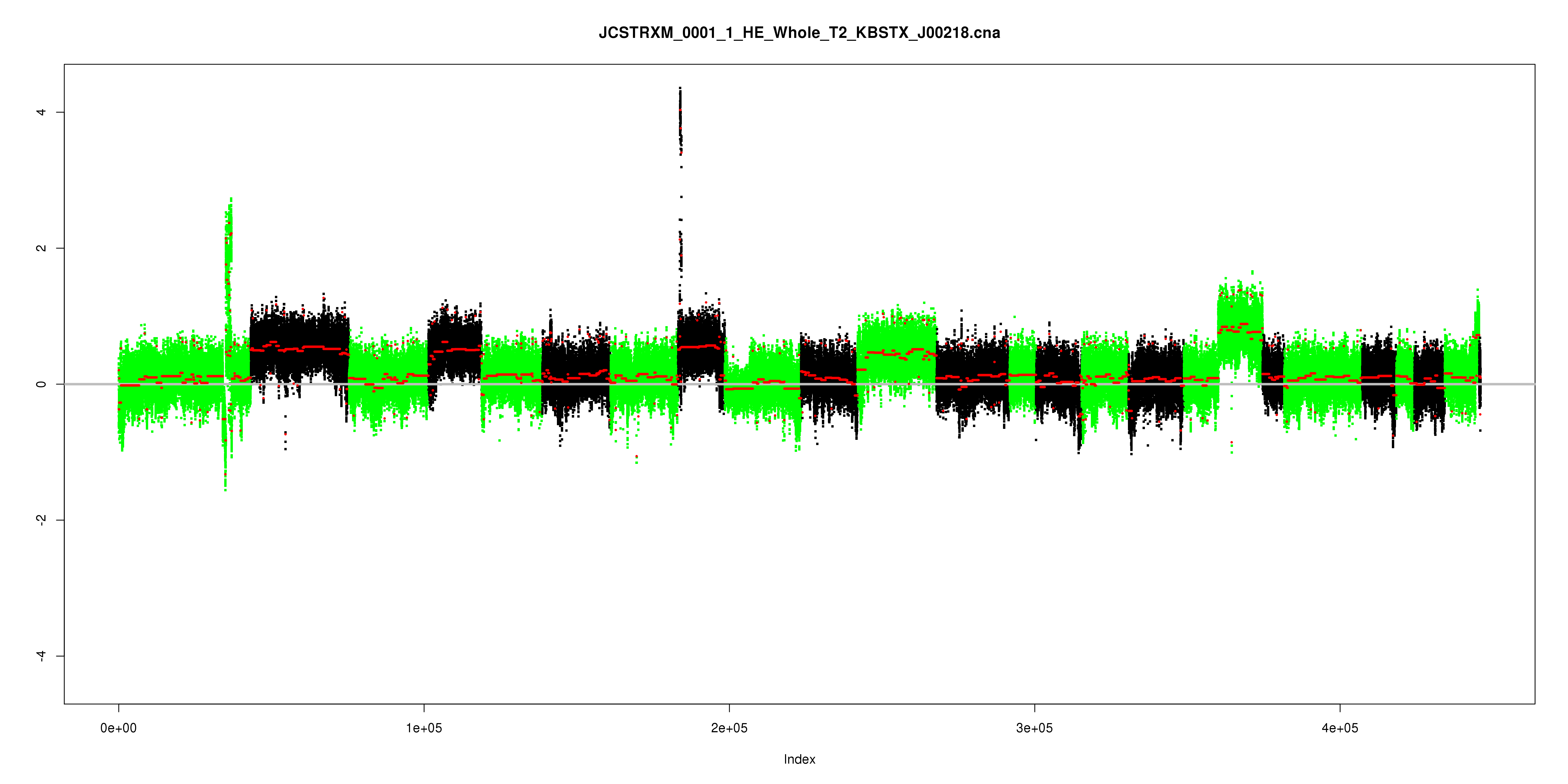

Supplement: Additional file 3: Figure S1. — Genome-wide chromosomal copy number plot outputs from 1A) copy number analysis, 1B) LOH and allelic imbalance analysis, 1C) segmentation algorithm copy number analysis, and 1D) ExomeCNV copy number analysis. 1A) chromosomal plots contain chromosomal map position in megabases on the X-axis, and the log2 fold change ratio information on the Y-axis. Regions of copy neutrality (ratios between log2FC -0.75 and +0.75) are black, regions of copy number gain (ratio > log2FC 0.75) are red, and regions of copy number loss (ratios < log2FC -0.75) are green. 1B) chromosomal plots contain chromosomal map position in megabases on the X-axis, and the B-allele frequency (BAF) on the Y-axis revealing chromosomal allelic imbalances. 1C) the copy number log2 fold change ratios (Y-axis) for each chromosome are colored alternately in green and black across the entirety of the genome map positions along the X-axis. 1D) chromosomal plots contain chromosomal map position in megabases on the X-axis, and the log2 fold change ratio information on the Y-axis derived from ExomeCNV. (ZIP 7726 kb) [file 12885_2016_3000_MOESM3_ESM.zip › Supplementary Figure 1CR4.jpg]

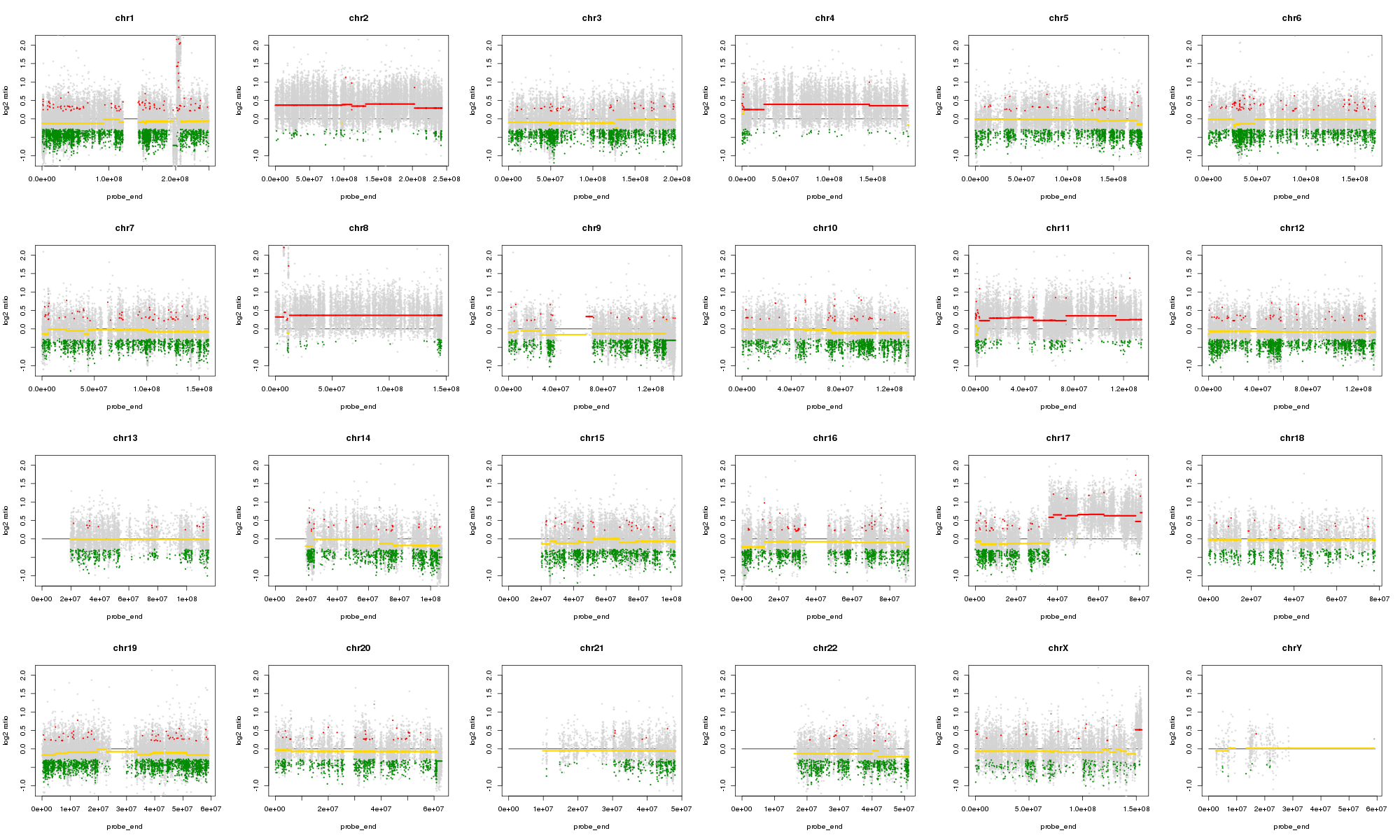

Supplement: Additional file 3: Figure S1. — Genome-wide chromosomal copy number plot outputs from 1A) copy number analysis, 1B) LOH and allelic imbalance analysis, 1C) segmentation algorithm copy number analysis, and 1D) ExomeCNV copy number analysis. 1A) chromosomal plots contain chromosomal map position in megabases on the X-axis, and the log2 fold change ratio information on the Y-axis. Regions of copy neutrality (ratios between log2FC -0.75 and +0.75) are black, regions of copy number gain (ratio > log2FC 0.75) are red, and regions of copy number loss (ratios < log2FC -0.75) are green. 1B) chromosomal plots contain chromosomal map position in megabases on the X-axis, and the B-allele frequency (BAF) on the Y-axis revealing chromosomal allelic imbalances. 1C) the copy number log2 fold change ratios (Y-axis) for each chromosome are colored alternately in green and black across the entirety of the genome map positions along the X-axis. 1D) chromosomal plots contain chromosomal map position in megabases on the X-axis, and the log2 fold change ratio information on the Y-axis derived from ExomeCNV. (ZIP 7726 kb) [file 12885_2016_3000_MOESM3_ESM.zip › Supplementary Figure 1DR4.jpg]

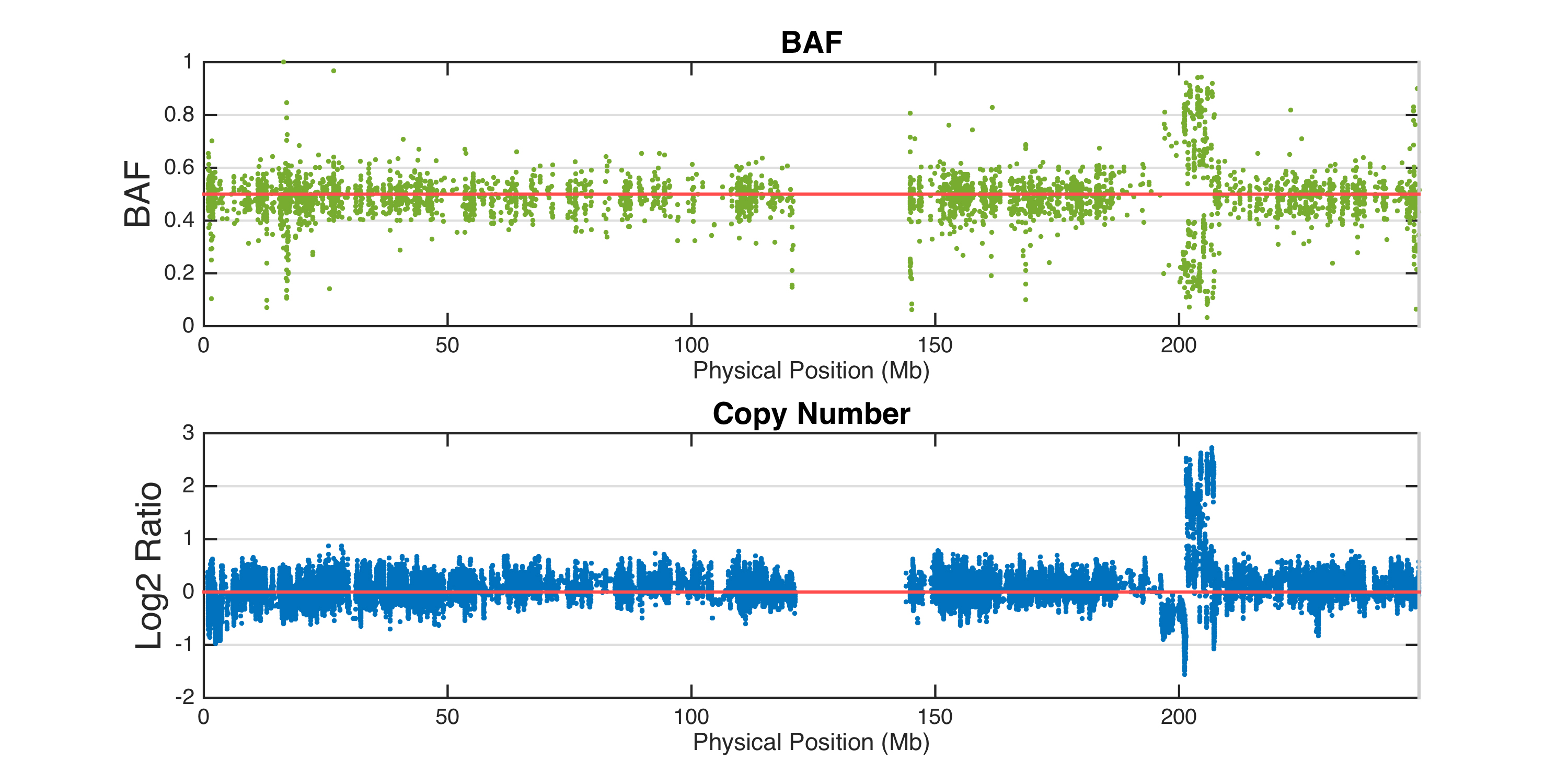

Supplement: Additional file 4: Figure S2. — Chromosome 1 copy number plot outputs illustrating 1q32 amplification. Plots represent outputs from (2A upper panel) LOH and allelic imbalance analysis, and (2A lower panel) copy number analysis. 2A upper panel, chromosome 1 plot with map position in megabases on the X-axis, and the B-allele frequency (BAF) on the Y-axis revealing chromosomal allelic imbalances. 2A lower panel, chromosome 1 plot with map position in megabases on the X-axis, and the log2 fold change ratio information on the Y-axis. 2B shows 10 megabase zoomed region of chromosome 1 (1q32) amplicon. 2B upper panel, chromosome 1 zoomed in plot with map position in megabases on the X-axis, and the B-allele frequency (BAF) on the Y-axis revealing chromosomal allelic imbalance at this region. 2B lower panel, chromosome 1 zoomed in plot with map position in megabases on the X-axis, and the log2 fold change ratio information on the Y-axis. The map postion related to the MDM4 gene locus is depicted as a purple line on each plot. (ZIP 1288 kb) [file 12885_2016_3000_MOESM4_ESM.zip › Supplementary Figure 2AR4.jpg]

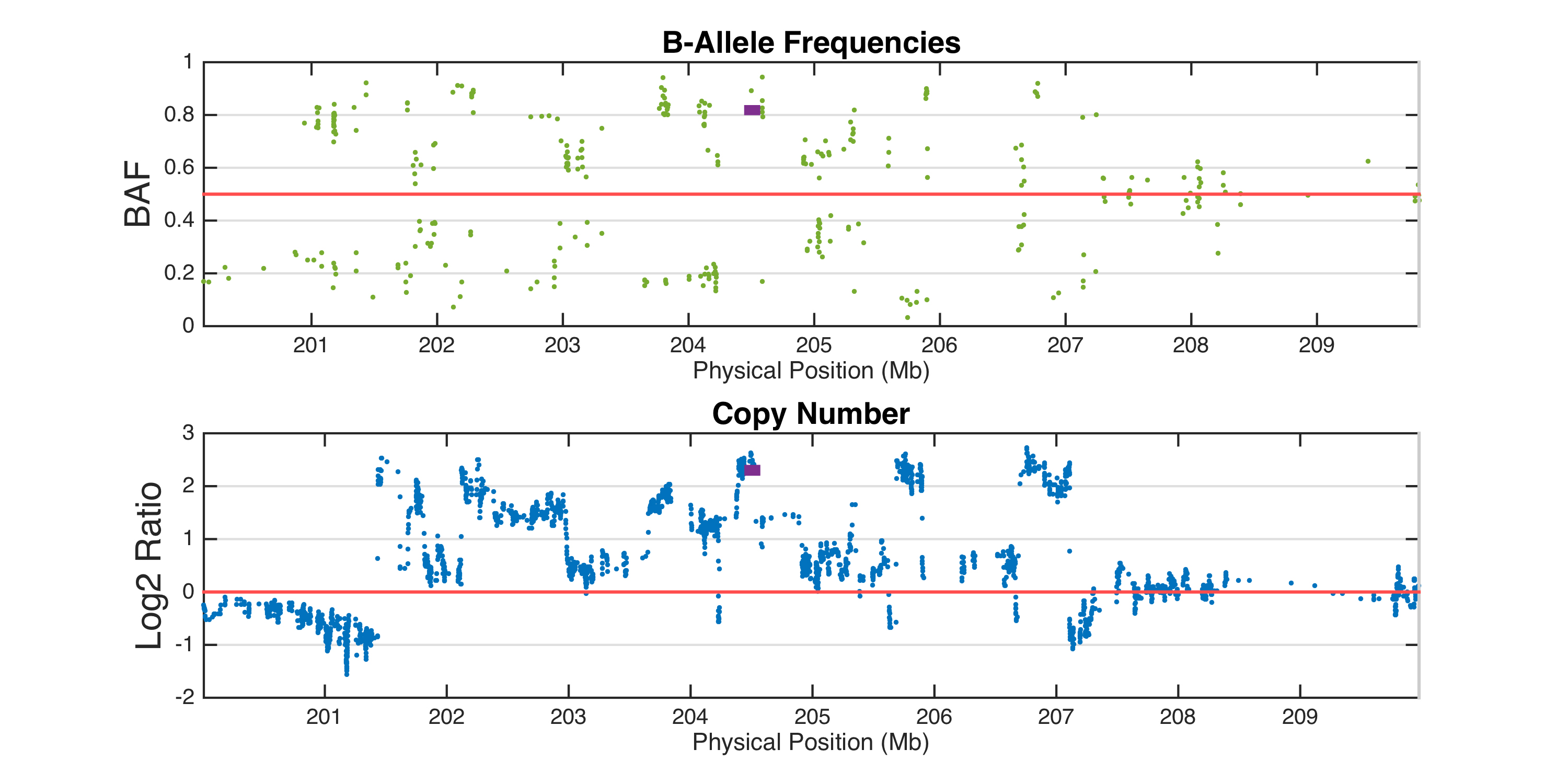

Supplement: Additional file 4: Figure S2. — Chromosome 1 copy number plot outputs illustrating 1q32 amplification. Plots represent outputs from (2A upper panel) LOH and allelic imbalance analysis, and (2A lower panel) copy number analysis. 2A upper panel, chromosome 1 plot with map position in megabases on the X-axis, and the B-allele frequency (BAF) on the Y-axis revealing chromosomal allelic imbalances. 2A lower panel, chromosome 1 plot with map position in megabases on the X-axis, and the log2 fold change ratio information on the Y-axis. 2B shows 10 megabase zoomed region of chromosome 1 (1q32) amplicon. 2B upper panel, chromosome 1 zoomed in plot with map position in megabases on the X-axis, and the B-allele frequency (BAF) on the Y-axis revealing chromosomal allelic imbalance at this region. 2B lower panel, chromosome 1 zoomed in plot with map position in megabases on the X-axis, and the log2 fold change ratio information on the Y-axis. The map postion related to the MDM4 gene locus is depicted as a purple line on each plot. (ZIP 1288 kb) [file 12885_2016_3000_MOESM4_ESM.zip › Supplementary Figure 2BR4.jpg]

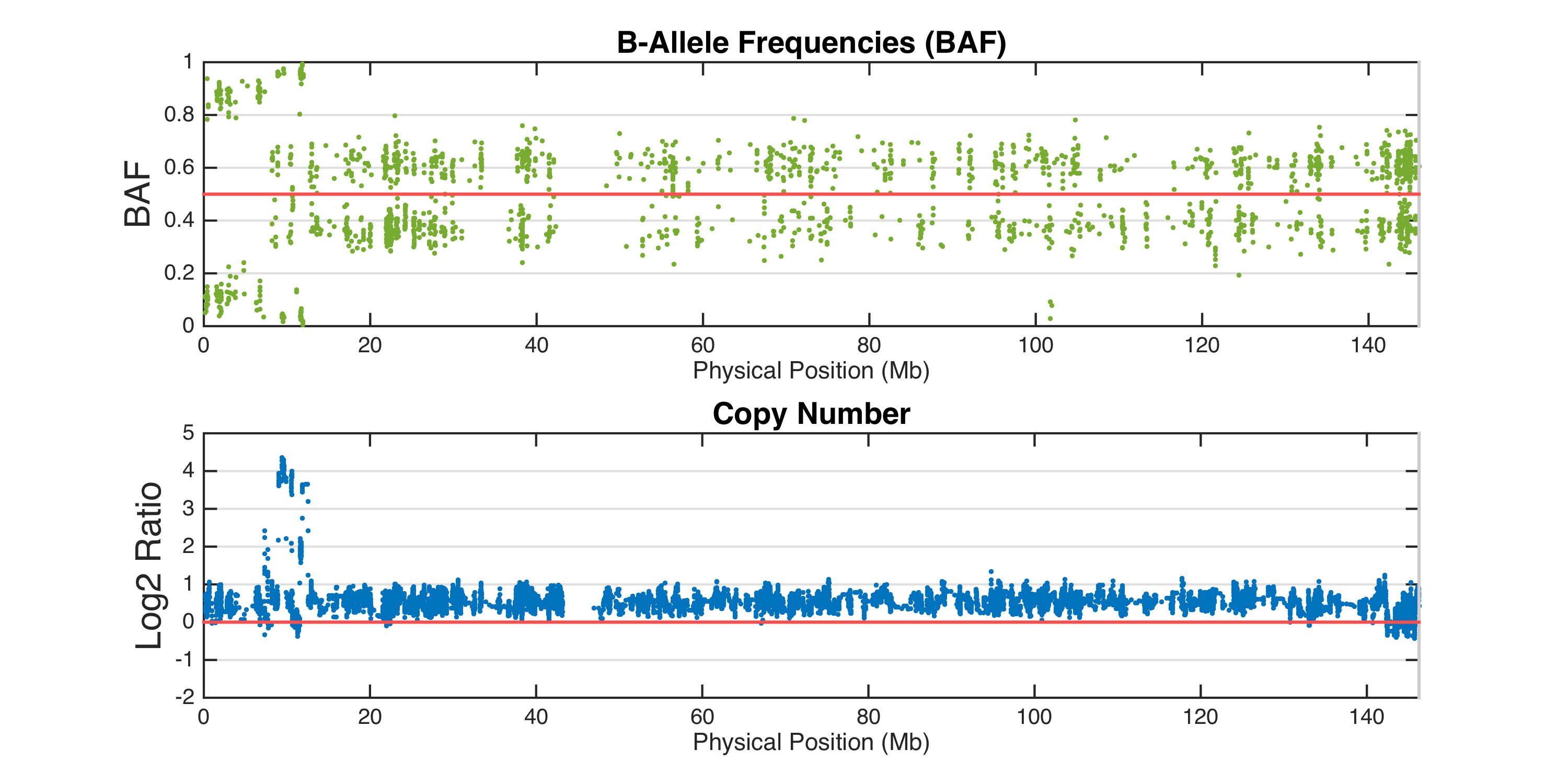

Supplement: Additional file 5: Figure S3. — Chromosome 8 copy number plot outputs illustrating 8p allelic imbalance and 8p23 amplification. Plots represent outputs from (upper panel) custom algorithm providing information on allelic imbalances, and (lower panel) copy number analysis. Upper panel, chromosome 8 plot with map position in megabases on the X-axis, and the B-allele frequency (BAF) on the Y-axis revealing chromosomal allelic imbalances. Lower panel, chromosome 8 plot with map position in megabases on the X-axis, and the log2 fold change ratio information on the Y-axis. (JPG 1099 kb) [file 12885_2016_3000_MOESM5_ESM.jpg]
